# Combination of Synovial Fluid IL-4 and Polymorphonuclear Cell Percentage Improves the Diagnostic Accuracy of Chronic Periprosthetic Joint Infection

**DOI:** 10.3389/fsurg.2022.843187

**Published:** 2022-03-09

**Authors:** Jiaxing Huang, Jiawei Wang, Leilei Qin, Bo Zhu, Wei Huang, Ning Hu

**Affiliations:** ^1^Department of Orthopedics, The First Affiliated Hospital of Chongqing Medical University, Chongqing, China; ^2^Orthopedic Laboratory of Chongqing Medical University, Chongqing, China

**Keywords:** synovial fluid, interleukin 4, polymorphonuclear cell percentage, chronic periprosthetic joint infection, diagnosis

## Abstract

**Background:**

Synovial fluid biomarkers have been found to improve the diagnosis of chronic periprosthetic joint infection (PJI); however, no “gold standard” exists yet. Interleukin-4 (IL-4) and polymorphonuclear cell (neutrophil) count in the synovial fluid are crucial in mediating local inflammation during bacterial infections and could be valuable biomarkers for PJI.

**Methods:**

This prospective study was conducted to investigate the diagnostic potential of synovial fluid IL-4 (SF-IL4) and polymorphonuclear cell percentage (SF-PMN%) for chronic PJI. A total of 110 patients who underwent revision arthroplasty between January 2019 and October 2020 were enrolled, and 11 patients were excluded. Of 99 patients, 43 were classified as having PJI and 56 as having aseptic failures according to the 2013 Musculoskeletal Infections Society criteria. In all patients, SF-IL4, SF-PMN%, serum C-reactive protein (CRP), and serum erythrocyte sedimentation rate (ESR) were quantified preoperatively. The diagnostic value for each biomarker was analyzed, and optimal cutoff values were calculated.

**Results:**

The patient demographics did not significantly vary. The area under the curve of SF-IL4 and SF-PMN% was 0.97 and 0.89, respectively, higher than that for serum ESR (0.72) and serum CRP (0.83). The combination of SF-IL4 and SF-PMN% provided higher specificity (97.0%) and accuracy (96.0%) when the cut-off values were 1.7 pg/mL and 75%, respectively.

**Conclusion:**

SF-IL4 is a valuable biomarker for chronic PJI detection, and the combination of SF-IL4 and SF-PMN% improved the diagnostic value of chronic PJI, and further studies are needed until its clinical application.

## Introduction

Periprosthetic joint infection (PJI) is a catastrophic complication after joint replacement surgery. Total knee arthroplasty (TKA) and total hip arthroplasty (THA) have low incidence rates of 0.3–2.4% and 0.8–2%, respectively (1, 2); however, PJI is the most common cause of revision for failed TKA and the third most common cause for revision for failed THA (3, 4). The cost of treatment of a PJI is three to four times higher than that for primary surgery (5). It is important to preoperatively distinguish between PJI and aseptic failure because it will determine the course of antibiotic therapy and surgical strategies.

Currently, there is no “gold standard” for PJI diagnosis (6). The diagnostic standards for PJI have been refined over the past decades (7–9). Serum C-reactive protein (CRP) and erythrocyte sedimentation rate (ESR) are highly sensitive markers of PJI, but their lack of specificity limits their diagnostic accuracy (10–12). In recent years, the use of synovial fluid (SF) biomarkers to diagnose PJI has attracted attention because of their low cost, ease of interpretation, and high accuracy (13, 14). Neutrophils are the first cells to produce a defensive response to bacterial infections; therefore, they are suitable candidates to achieve high sensitivity and specificity for PJI detection (15). SF-PMN% has been the basic indicator of SF analysis, but the cut-off value is still controversial (14, 16). Therefore, more valuable biomarkers to detect chronic PJI are needed.

IL-4 is involved in acute and chronic bacterial defense inflammatory responses, but the diagnostic value for chronic PJI is not yet known. IL-4 promotes immunoglobulin isotype switching and regulates the function of macrophages mainly via the Stat6 pathway (17). Serum IL-4 level is upregulated in bacterial infection-induced systemic inflammatory response syndrome and can be a good predictor of infection-related mortality risk (18). Serum IL-4 can be used as an immunological marker for diagnosing active tuberculosis and for monitoring the efficacy of antituberculosis therapy (19). Local administration or expression of IL-4 enhanced the pulmonary clearance of *Pseudomonas aeruginosa in vivo* and decreased mortality following infection (20). In joint aspiration, IL-4 was first secreted by synovial mast cells and then by T helper 2 (Th2) cells (21). Previous studies have demonstrated that the level of IL-4 is elevated in the SF during bacterial infection (6, 22). Therefore, we hypothesized that SF-IL4 may be used as a useful biomarker for chronic PJI detection.

This study was conducted to (1) explore and set an optimal cut-off value for serum CRP, ESR, SF-IL4, and SF-PMN% for chronic PJI diagnosis and (2) improve the diagnostic efficiency of chronic PJI by combining SF-IL4 with other biomarkers.

## Materials and Methods

This prospective study protocol was approved by the institutional ethics board of the First Affiliated Hospital of Chongqing Medical University. Informed consent was obtained from every patient. Patients who underwent revision TKA or THA between January 2019 and October 2020 were enrolled in this study. Patients were divided into chronic PJI group and aseptic failure group based on the 2013 Musculoskeletal Infections Society criteria (2013 MSIS) (Table 1) (8). Chronic PJI was defined as the occurrence of PJI symptoms more than 6 weeks after the primary implantation (23, 24). Aseptic failures included aseptic loosening, wear, instability, dislocation, adverse local tissue reactions, and metal allergic reactions (9). To rule out interference with other possible preconditions associated with elevated inflammatory factors, patients with (1) infections of other organs, including pneumonia and urinary tract infection, (2) active rheumatoid arthritis, ankylosing spondylitis, and gouty arthritis, or (3) malignant tumors were excluded.

**Table 1:** The Musculoskeletal society 2013 criteria for defining periprosthetic joint infection.

**Periprosthetic joint infection is present if one of two major criteria or three of five minor criteria exists**	
Major criteria	1. A sinus tract communicating with the joint; or 2. Two positive periprosthetic cultures with phenotypical identical organisms
Minor criteria	1. Elevated serum C-reactive protein (CRP) AND erythrocyte sedimentation rate (ESR); or 2. Elevated synovial fluid white blood cell (WBC) count OR ++ change on leukocyte esterase strip; or 3. Elevated synovial fluid polymorphonuclear neutrophil percentage (PMN%); or 4. Positive histological analysis of periprosthetic tissue; or 5. A single positive culture

Data on patient demographics (age, gender, preoperative diagnosis [PJI or aseptic failure and joint involved: hip or knee]) and the survival time of primary implantation were collected. Before revision surgery, peripheral venous blood (3 mL) was withdrawn to test serum ESR and CRP levels. SF (3–4 mL) was sampled for analyzing SF-IL4, SF-PMN%, and culture (48-h routine culture and 14-day prolonged culture; at least three intraoperative tissues were cultured). All biochemical assays were performed at the biochemistry laboratory of the biology technical platform in our institution.

### Statistical Analysis

Data were analyzed using SPSS 26.0 (IBM Corporation, TX). Continuous data with a non-normal distribution are presented as the median and interquartile range (IQR). Mann-Whitney U test was used to analyze the statistical significance, and the chi-square test was used to compare the sensitivity and specificity of laboratory test data and categorical data (age, gender, joint involved). P < 0.05 (two-tailed) was considered to indicate a statistically significant difference. Receiver operating characteristic (ROC) curves and the area under the curve (AUC) were calculated using MedCalc 19.0.7 (Ostend, Belgium). The sensitivity, specificity, positive predictive value (PPV), and negative predictive value (NPV) were estimated for tested markers. The optimal cut-off value for each maker was computed using the maximized Youden index (sensitivity+specificity-1) method. A higher diagnostic odds ratio (DOR) indicated better discriminatory strength (25).

## Results

### Baseline Data of the Two Groups Did Not Significantly Differ

Of the 110 patients initially enrolled, six patients were excluded due to “dry aspiration,” four patients with active rheumatoid arthritis, and one patient with acute PJI. Finally, 99 patients were included in the study, with 43 (43.4%) in the chronic PJI group and 56 (56.6%) in the aseptic failure group. The baseline characteristics of the two groups were similar (Table 2) with no significant difference in the demographics (*P* > 0.05).

**Table 2:** Demographic data for the study population.

**Characteristic**	**Chronic PJI** **(*N* = 43)**	**Aseptic failure** **(*N* = 56)**	***P*-value**
Age (year)	70.58 ± 5.26	68.98 ± 6.05	0.17^*^
**Gender**			
Male	22 (51.2%)	27 (48.2%)	0.77^*^
Female	21 (48.8%)	29 (51.8%)	
**Joint type**			
Hip	17 (39.5%)	31 (55.4%)	0.12^*^
Knee	26 (60.5%)	25 (44.6%)	
BMI (kg/m^2^)	22.74 ± 3.91	23.21 ± 4.66	0.59^**#**^
Survival time of implantation (month)	67.91 ± 29.06	76.82 ± 21.55	0.08^**#**^

* *Chi-square test*,

# *independent Student's t-test; PJI, periprosthetic joint infection*.

### SF-IL4 Had Higher Diagnostic Power Than Serum ESR and CRP Levels

As shown in Table 3, the median value of serum ESR (35.00 mm/h [7.20–120.00 mm/h]) was higher in the chronic PJI group than in the aseptic failure group (22.0 mm/h [2.00–58.00 mm/h]; *P* = 0.001). Similarly, serum CRP level (21.4 mg/L [5.80–91.20 mg/L]) was higher in the chronic PJI group than in the aseptic failure group (6.75 mg/L [1.25–28.00]; *P* < 0.001). Results showed that the median level of SF-IL4 in the chronic PJI group is higher than that in the aseptic group (3.30 vs. 1.10 pg/mL, *P* < 0.0001). Finally, the median SF-PMN% was higher in the PJI group (87.58%) than in the aseptic failure group (56.95%), with statistical significance (*P* < 0.001).

**Table 3:** Analysis of single markers in patients with chronic PJI and aseptic failure.

**Marker**		**Chronic PJI** **(*N* = 43)**	**Aseptic failure** **(*N* = 56)**	***P*-value**
ESR (mm/h)	Range	7.20–120.00	2.00–58.00	
	Median	35.00	22.00	0.001^#^
	P25, P75	22.00; 50.00	17.50; 32.00	
	Mean ± SD	42.10 ± 26.23	24.38 ± 12.80	
CRP (mg/L)	Range	5.80–91.20	1.25–28.00	
	Median	21.40	6.75	<0.001^#^
	P25, P75	13.20; 33.40	3.29; 16.00	
	Mean ± SD	27.31 ± 20.02	9.81 ± 7.75	
SF-IL4 (pg/mL)	Range	1.20–14.10	0.09–2.88	
	Median	3.30	1.10	<0.001^#^
	P25, P75	2.20; 9.7	0.55; 1.28	
	Mean ± SD	5.57 ± 4.37	1.00 ± 0.53	
SF-PMN%	Range	60.32–96.65	23–89.20	
	Median	87.58	56.95	<0.001^#^
	P25, P75	83.50, 90.50	53.40, 56.95	
	Mean ± SD	86.15 ± 7.31	59.30 ± 15.53	

*SD, standard deviation*;

# *Mann-Whitney U test*.

**Figure 1:**
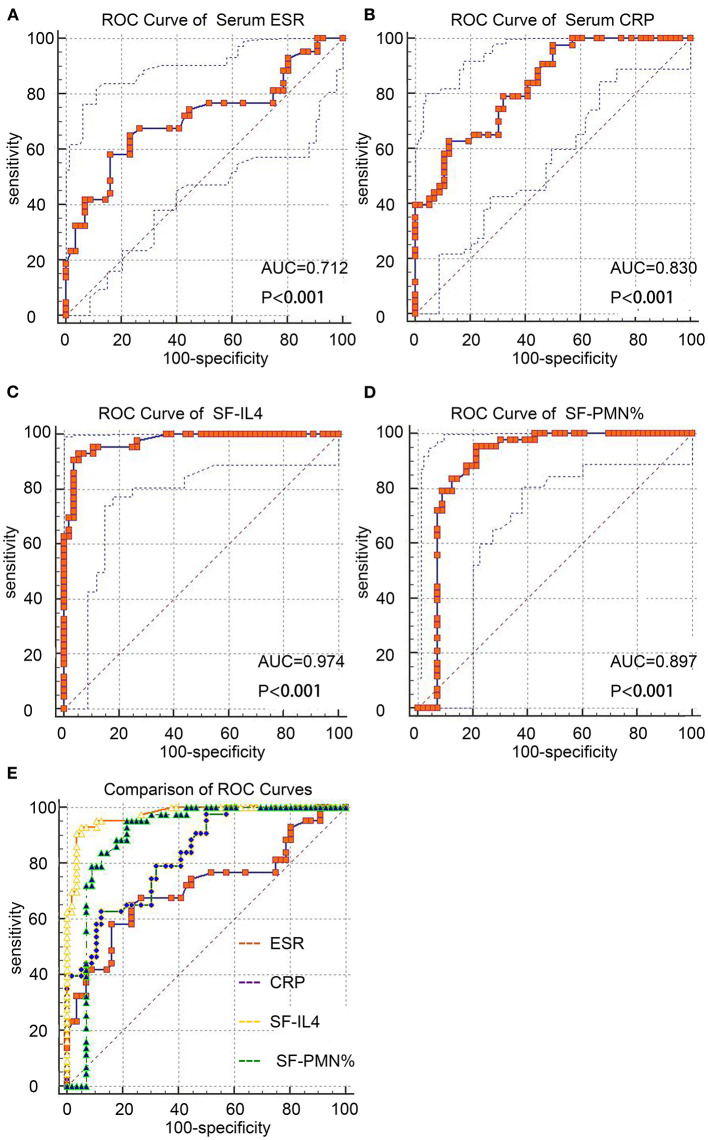
ROC curves of the studied markers for chronic PJI diagnosis. **(A–D)** ROC curves of serum ESR, CRP, SF-IL4, and SF-PMN%. **(E)** Comparison of these four markers.

ROC curves were used to measure the discriminatory strength of the indicators (Figures 1A–D). The AUC of SF-IL4 (0.97 [95% CI, 0.92–0.99]) was higher than that of serum ESR (0.72 [95% CI, 0.62–0.84]; *P* = 0.0004), serum CRP (0.83 [95% CI, 0.74–0.90]; *P* <0.0001), and SF-PMN% 0.89 [95% CI, 0.82–0.95]; *P* = 0.053) (Figure 1E). The sensitivity, specificity, PPV, NPV, +LR, –LR, and DOR of these markers are in Table 4. The optimal cut-off value for SF-IL4 of 1.7 pg/mL achieved a sensitivity of 93.02% (95% CI, 80.9–98.5%), specificity of 94.64% (95% CI, 85.1–98.9%), and high PPV (93.0%), NPV (94.6%), and DOR (248).

**Table 4:** AUC, cut-off value, and diagnostic value of each marker for chronic PJI.

**Test**	**CRP (mg/L)**	**ESR (mm/h)**	**CRP (mg/L)**	**ESR (mm/h)**	**SF-IL4 (pg/mL)**	**SF-PMN%**
AUC (95% CI)			0.83 (0.74–0.90)	0.72 (0.62–0.84)	0.97 (0.92–0.99)	0.89 (0.82–0.95)
Cut-off level	10	30	18	34	1.7	75%
Sensitivity (95% CI)	81.4	67.4	62.79 (46.7–77.0)	58.14 (42.1–73.0)	93.02 (80.9–98.5)	95.35 (84.2–99.4)
Specificity (95% CI)	58.9	62.6	87.50 (75.9–94.8)	83.93 (71.7–92.4)	94.64 (85.1–98.9)	78.57 (65.6–88.4)
PPV	–	–	79.4 (65.0– 88.9)	73.5 (59.2–84.2)	93.0 (81.6–97.6)	77.4 (67.3–85.0)
NPV	–		75.4 (67.0– 82.10)	72.3 (64.3 −79.1)	94.6 (85.6–98.1)	95.7 (85.0–98.8)
+LR	–	–	5.02 (2.40– 10.40)	3.62 (1.9–6.9)	17.36 (5.8–52.4)	4.45 (2.7–7.4)
–LR	–	–	0.43 (0.3–0.6)	0.50 (0.3– 0.7)	0.07 (0.02–0.2)	0.06 (0.02–0.2)
DOR	–	–	11.67	7.24	248.00	74.17
Accuracy	58.6	50.5	72.7	68.7	92.9	84.8

*AUC, area under the curve; PPV, positive predictive value; NPV, negative predictive value; +LR, positive likelihood ratio; –LR, negative likelihood ratio; DOR, diagnostic odds ratio*.

### Combination of SF-IL4 With SF-PMN% Had Improved Diagnostic Value for Chronic PJI

Next, we combined SF-IL4 with other biomarkers (Table 5). When the cut-off values of SF-IL4 and SF-PMN% were met at the same time, the specificity increased to 97% and accuracy increased to 96%, but the sensitivity decreased to 91% for chronic PJI diagnosis.

**Table 5:** Diagnostic value of different combinations of markers for chronic PJI diagnosis.

**Combination**	**SF-IL4>1.7 pg/mL** **+ CRP >18 mg/L**	**SF-IL4 >1.7 pg/mL** **+ SF-PMN% >75%**	**CRP >18 mg/L + SF-IL4** **>1.7 pg/mL+** **SF-PMN% >75%**
Sensitivity	0.88	0.91	0.53
Specificity	0.98	0.97	0.98
PPV	0.96	0.94	0.95
NPV	0.75	0.92	0.73
Accuracy	80.1%	96%	78.8%

*PPV, positive predictive value; NPV, negative predictive value*.

## Discussion

PJI is still a catastrophic complication of arthroplasty. Chronic PJI patients have poorer function score, lower quality of life, and significantly increased risk of short-term mortality (26–28). The diagnosis of chronic PJI relies on clinical symptoms, physical examination, biomarkers examination, and radiological examination. Because chronic PJI is a type of encapsulated and low-grade infection, it usually causes less extensive systemic inflammatory reactions, sometimes resulting in negative laboratory test results (29). Therefore, the diagnosis of chronic PJI is a challenge, especially without a “gold standard” (30).

SF interleukins play an important role in implant-associated infection. TNF-α, IL-1, and IL-6 are pro-inflammatory cytokines essential for initiating an inflammatory response to infection (31). IL-4 is an anti-inflammatory cytokine that participates in regulating chronic infection and immune processes (32). IL-4 regulates the ratio of Th1/Th2 lymphocyte subtypes in chronic infections (17). It can inhibit the development of biofilms in chronic infections of *Staphylococcus aureus* and promote spontaneous infection clearance (33). IL-4 induces the macrophage switch from the M1 to M2 phenotype, which inhibits osteoclast differentiation (34, 35), and promotes the differentiation of B cells and plasma cells, participates in humoral immunity, and exhibits anti-infective properties (36, 37).

Similarly, SF-IL4 has shown promising potential for PJI diagnosis. Gollwitzer et al. reported that SF-IL4 has 93% sensitivity and 85% specificity in PJI, making it better than other serum markers and SF cytokines such as IL-1β and IL-6 (6). However, their study only enrolled patients with *S. aureus* infection, which is more virulent. Therefore, the cut-off value for SF-IL4 was 7.79 pg/mL in their study, much higher than our cut-off value (1.7 pg/mL). Fröschen et al. found that combining SF-IL2, SF-IL4, SF-IL5, SF-IL6, SF-IL12, and granulocyte-macrophage colony-stimulating factor can achieve 100% sensitivity and 88.9% specificity, but these are not universal values as it is expensive to quantify all these markers (38). Consistent with previous studies, we found that the median level of SF-IL4 in the chronic PJI group was 3.30 pg/mL. When the cut-off value was set as 1.7 pg/mL for SF-IL4, the highest sensitivity (93.02 [95% CI, 80.9–98.5%]), specificity (94.64% [95% CI, 85.1–98.9%]), and diagnostic accuracy (94.6% [95% CI, 85.1–98.9%]) were obtained compared with values for serum ESR, serum CRP, and SF-PMN%.

Synovial leukocyte analysis is the basis of the SF test, Synovial fluid leukocyte counts and differentiation ratios have been widely proposed and discussed as secondary diagnostic criteria for chronic PJI. Differences in thresholds exist between different institutions and counting methods. Leukocyte counts are more strongly affected by many factors, especially the use of antibiotics before joint aspiration (15, 39–41) and metallosis, while neutrophil percentages are more stable with infection An SF-PMN% more than 80% has been recommended for the diagnosis of chronic PJI (>6 weeks after surgery) in the 2013 MSIS consensus (8). Zahar et al. found that an SF-PMN% cut-off of 66.1% achieved a sensitivity of 80.6% and specificity of 83.3% for chronic PJI (42). Higuera et al. used an SF-PMN% cut-off of 80% and found the sensitivity and specificity of chronic hip PJI as 92.1 and 85.8%, respectively (16). In the present study, when the optimal cut-off value of SF-PMN% was 75%, the AUC of diagnosing chronic PJI was 0.89 (95% CI, 0.82–0.95), with higher sensitivity of 95.35% (95% CI, 84.2–99.4%) but relatively decreased specificity of 78.57% (95% CI, 65.6–88.4%), respectively.

The 2013 MSIS consensus recommends a CRP cut-off value of 10 mg/L, and at this cut-off, the sensitivity and specificity were 81.4 and 58.9%, respectively, and the diagnostic accuracy was only 58.6% in our study. When the cut-off value of serum ESR was 30 mm/h, the sensitivity, specificity, and accuracy were 67.4, 62.6, and 50.5%, respectively. The unacceptably low sensitivity was coupled with a high number of false negatives. When we set the cut-off values of serum CRP and ESR levels as 18 mg/L and 34 mm/h, respectively, the detection accuracy for chronic PJI improved to 72.7 and 68.7%, respectively.

No single test could provide 100% accuracy for PJI diagnosis. Therefore, a combination of different indicators, with different sensitivity and specificity values, should be used to confirm or rule out the infection under high clinical suspicion (43–46). We found that when the SF-IL4 is >1.7 pg/mL and the SF-PMN% was more than 75%, the specificity and accuracy improved to 97 and 96%, respectively, for chronic PJI diagnosis. However, the combined diagnosis had high specificity but reduced diagnostic sensitivity, which may lead to missed diagnoses in some patients.

This study had some limitations. First, we did not adopt the latest 2018 ICM modified PJI diagnostic criteria, which includes new markers such as serum D-dimer and a scoring system, which is validated to have a higher sensitivity of 97.7% and specificity of 99.5% (47); however, the application of D-dimer for PJI is still controversial (48, 49). Second, we excluded patients diagnosed with active inflammatory arthritis at the time of admission, including rheumatoid arthritis, ankylosing spondylitis, and gouty arthritis. As these patients usually have higher serum ESR, serum CRP, SF-WBC count, and SF-PMN%, the inclusion of this group of patients will affect the accuracy of our results (39), and excluding these patients limits the clinical application of our results due to the limited sample size. Therefore, multicenter studies with larger sample sizes are required to produce more reliable results.

## Conclusion

When we set the cut-off value as 1.7 pg/mL, SF-IL4 achieved a higher sensitivity of 93.02 and specificity of 94.64% than serum ESR, serum CRP, and SF-PMN%. The combined measurement of SF-IL4 and SF-PMN% improved the specificity to 97% and the diagnostic accuracy for chronic PJI to 96%. However, the cut-off value of SF-IL4 for chronic PJI detection is not yet consensual, requiring more research data to support our conclusion.

## Data Availability Statement

The raw data supporting the conclusions of this article will be made available by the authors, without undue reservation.

## Ethics Statement

The studies involving human participants were reviewed and approved by the Institutional Ethics Board of the First Affiliated Hospital of Chongqing Medical University (Ethics approved number: 20187101). Written informed consent to participate in this study was provided by the participants' legal guardian/next of kin. Written informed consent was obtained from the individual(s) for the publication of any potentially identifiable images or data included in this article.

## Author Contributions

NH and WH conceived and designed the study. JH, JW, and BZ analyzed and interpreted the data. JH drafted the article. NH and WH critically revised the paper. All of the authors approved the final submitted version.

## Funding

This research was supported by the Chinese National Natural Science Foundation (grant number: 82072443) and Chongqing Medical Scientific Research Project (joint project of Chongqing Health Commission and Science and Technology Bureau) (grant number: 2019ZDXM014).

## Conflict of Interest

The authors declare that the research was conducted in the absence of any commercial or financial relationships that could be construed as a potential conflict of interest.

## Publisher's Note

All claims expressed in this article are solely those of the authors and do not necessarily represent those of their affiliated organizations, or those of the publisher, the editors and the reviewers. Any product that may be evaluated in this article, or claim that may be made by its manufacturer, is not guaranteed or endorsed by the publisher.

## Abbreviations

PJI: periprosthetic joint infection
ESR: erythrocyte sedimentation rate
CRP: C-reactive protein
SF-IL4: synovial fluid interleukin 4
SF-PMN%: synovial fluid polymorphonuclear cell neutrophil percentage
PPV: positive predictive value
NPV: negative predictive value
+LR: positive likelihood ratio
-LR: negative likelihood ratio
DOR: diagnostic odds ratio
ROC: receiver operating characteristic
AUC: area under the curve.
